# Copper-Associated Oxidative Stress Contributes to Cellular Inflammatory Responses in Cystic Fibrosis

**DOI:** 10.3390/biomedicines9040329

**Published:** 2021-03-24

**Authors:** Amal Kouadri, Johanna Cormenier, Kevin Gemy, Laurence Macari, Peggy Charbonnier, Pierre Richaud, Isabelle Michaud-Soret, Nadia Alfaidy, Mohamed Benharouga

**Affiliations:** 1 Institut National de la Santé et de la Recherche Médicale U1292, Biologie et Biotechnologie Pour la Santé, 38000 Grenoble, France; amal.kouadri.phd@gmail.com (A.K.); cormenierj@gmail.com (J.C.); Kevin.GEMY@cea.fr (K.G.); 2 Commissariat à l’Energie Atomique et Aux Energies Alternatives (CEA), 38000 Grenoble, France; laurence.macari@cea.fr (L.M.); peggy.charbonnier@cea.fr (P.C.); isabelle.michaud-soret@cea.fr (I.M.-S.); 3 Université Grenoble Alpes (UGA), 38043 Grenoble, France; 4 Centre National de la Recherche Scientifique (CNRS), LCBM-UMR 5249, 38000 Grenoble, France; 5 CEA, CNRS, Institut de Biosciences et Biotechnologies d’Aix-Marseille (BIAM), Université Aix-Marseille, UMR 7265, CEA Cadarache, 13108 Saint-Paul-lez Durance, France; richaudp@gmail.fr

**Keywords:** inflammation, cystic fibrosis, oxidative stress, CFTR, copper, lung

## Abstract

Cystic fibrosis (CF) is caused by mutations in the gene encoding the CF Transmembrane Conductance Regulator (CFTR), an apical chloride channel. An early inflammation (EI) in the lung of CF patients occurring in the absence of any bacterial infection has been reported. This EI has been proposed to be associated with oxidative stress (OX-S), generated by deregulations of the oxidant/antioxidant status. Recently, we demonstrated that copper (Cu), an essential trace element, mediates OX-S in bronchial cells. However, the role of this element in the development of CF-EI, in association with OX-S, has never been investigated. Using healthy (16HBE14o-; HBE), CF (CFBE14o-; CFBE), and corrected-wild type CFTR CF (CFBE-wt) bronchial cells, we characterized the inflammation and OX-S profiles in relation to the copper status and CFTR expression and function. We demonstrated that CFBE cells exhibited a CFTR-independent intrinsic inflammation. These cells also exhibited an alteration in mitochondria, UPR (Unfolded Protein Response), catalase, Cu/Zn- and Mn-SOD activities, and an increase in the intracellular content of iron, zinc, and Cu. The increase in Cu concentration was associated with OX-S and inflammatory responses. These data identify cellular Cu as a key factor in the generation of CF-associated OX-S and opens new areas of investigation to better understand CF-associated EI.

## 1. Introduction

Cystic fibrosis (CF) is an autosomal recessive disease characterized by viscous mucus [1] and abnormal ion transport across the apical plasma membrane (PM) of the gastrointestinal and the pulmonary epithelia [1]. CF is caused by mutations in the *CFTR* gene that encodes for CF Transmembrane Conductance Regulator (CFTR) protein, an apical transmembrane cAMP-regulated chloride (Cl^–^) channel [2]. Currently, there are more than 2000 mutations identified in the *CFTR* gene and classified according to their consequences on the CFTR activity, expression, or localization (http://www.genet.sickkids.on.ca/cftr/, accessed on 14 January 2021).

The most common CF mutation corresponds to a deletion of a phenylalanine (F) at position 508, F508del-CFTR. This mutation causes an abnormal conformation to F508-del-CFTR, leading to its recognition, retention, and degradation at the endoplasmic reticulum (ER) compartment [3]. However, even if the gating of the channel is affected by the F508 deletion, the mutated protein retains some functionality as a Cl^–^ channel [4]. The absence of a functional CFTR at the plasma membrane deregulates ions and water flux, leading to dehydrated secretion, thick mucus, reduced clearance of inhaled particles, including bacteria. This also leads to persistent infection and chronic inflammation, two major causes of the severe morbidity and mortality in CF population [1,5]. The CF airway contains large concentrations of several pro-inflammatory mediators, including tumor necrosis-α (TNFα), interleukin (IL)-1β, IL-6, IL-8, and IL-17 [5,6]. Importantly, secretion of these inflammatory mediators has been reported to be influenced by alterations in CFTR function [7]. An activation of the pro-inflammatory nuclear factor-κB (NF-κB) has also been reported in CF-Knockout mice [8]. The CF airway epithelium is also characterized by a decrease in the secretion of anti-inflammatory mediators, such as IL-10 [9]. Importantly, even in the absence of bacterial or viral pathogens, exacerbated inflammation has been observed in the respiratory tract of CF infants [10,11,12,13,14]. The origin of this inflammation has been a matter of debate and appeared to be the consequence of a hyperactivation of the NF-κB transcription factor and an ER retention of the CFTR-F508del [15].

Nevertheless, CFTR correction in CF epithelial cells did not reverse the levels of secreted cytokines, suggesting an intrinsic activation of inflammatory processes in these cells [16,17].

The inflammatory response in CF is complex, and involved different stimuli, including oxidative stress (OX-S) [18,19]. OX-S is defined as a disequilibrium in the balance between pro- and anti-oxidative factors [20]. The prominent of the pro-oxidants responses manifests by an increase in the levels of the reactive oxygen species (ROS) [20]. In CF, ROS levels are increased and participate in the injury of the lung by overwhelming endogenous antioxidant defenses [21,22].

The disequilibrium between pro- and anti-oxidant response causes the release of inflammatory mediators that damage the epithelial cell surface, along with an impairment of bacterial clearance [21,22]. The link between CF-associated OX-S and CFTR defects has previously been reported [22]. ER retention of unfolded CFTR-delF508 protein has been associated with the activation of the UPR (Unfolded Protein Response), and ER calcium expansion [23]. Additionally, CFTR dysfunction has been associated with an innate defect in the metabolism of glutathione (GSH), characterized by low GSH levels in the bronchial epithelial liquid [24]. Moreover, low activity of copper-(Cu) and zinc-(Zn) dependent superoxide dismutase (Cu/Zn-SOD) and mitochondrial dysfunction have been observed in cells with abnormal CFTR expression [25,26]. Importantly, all these systems are sensitive to copper homeostasis. This metal acts as an activator for Cu/Zn-SOD, as a natural substrate for the chelation by GSH, and as an electron source for mitochondrial activity [27]. In addition, copper is also an important trace element for numerous cell functions. However, it becomes toxic when its cellular homeostasis is disrupted. Copper-induced oxidative stress has been documented in vitro and in vivo [27], yet in vitro investigations on metal-induced oxidative stress, in relation to CF inflammation and CFTR expression and activity, have not been conducted in bronchial epithelial cells.

In the present study, we characterized three major processes of the development of the inflammation in CF bronchial cells, namely (i) oxidant and antioxidant statuses in healthy bronchial epithelial (HBE), CF bronchial epithelial (CFBE), and CFBE-wt cells; (ii) the inflammation profiles, and (iii) the role of CFTR and copper in redox balance and inflammation.

We demonstrated that CF bronchial epithelial cells exhibit, (i) elevated levels of ROS and catalase activity, (ii) decreased Cu/Zn- and Mn-SOD activity, and (iii) increased copper, iron, and zinc concentrations. Furthermore, we characterized the relationship between inflammation and the OX-S and unmasked the role of copper in CF-associated inflammatory processes.

## 2. Material and Methods

### 2.1. Cell Culture

The experiments were performed using human bronchiolar epithelial cell lines: 16HBE14o- (abbreviated as HBE), expressing wild-type CFTR; CFBE41o-derived from a CF patient (abbreviated as CFBE), homozygous for the dF508 mutation (dF508/dF508); CFBE-wtCFTR (abbreviated as CFBE-wt), CFBE41o-cells stably transfected with wt-CFTR protein (a generous gift from Dieter Gruenert, University of California at San Francisco, CA, USA). The HBE cells were used in this study because they have the capacity to form monolayer cells that are similar to the bronchial tissue [28].

The cells were grown in Eagle’s minimal essential medium (EMEM) (Thermofisher, France) supplemented with 10% fetal bovine serum (Biowest, France) at 37 °C under 5% CO_2_. The cells were grown in plastic dishes coated with an extracellular matrix containing fibronectin, collagen, and bovine serum albumin. Where indicated, cells were treated with a vehicle; with different concentrations of copper sulfate (CuSO_4_); of bathocuproine disulphonate (BCS), a copper chelator; or with the CFTR chloride channel inhibitor, CFTRinh-172 [29] for the indicated times. Copper was tested from 50–150 µM. The concentration 100 µM was used in our experiments as it led to a significant increase in the intracellular copper concentration after 24 h, without causing significant harmful effects [28].

### 2.2. Measurement of the cAMP-Stimulated Iodide Conductance of the Plasma Membrane

The plasma membrane cAMP-dependent halide conductance of tested cells was determined using iodide efflux technique, as previously described [30]. Iodide efflux was initiated by replacing the loading buffer with efflux medium (composed of 136 mM nitrate). The extracellular medium was replaced every minute with the efflux buffer (1 mL). After a steady state was reached, the intracellular cAMP level was raised by agonists (10 µM forskolin, 0.2 mM CTP-cAMP, and 0.2 mM isobutyl-methyl xanthane) to achieve maximal phosphorylation of the CFTR protein. The collection of the efflux medium resumed for an additional 6–9 min. The amount of iodide in each sample was determined with an iodide-selective electrode (Orion), and the cAMP-I efflux peaks were plotted.

### 2.3. Extraction of Total RNA and Reverse Transcription

Total RNA was extracted from the cells according to the manufacturer’s protocol (RNAgents; Promega, Charbonnières-les-Bains, Marnes-la-Coquette, France). An amount of 1 µg of total RNA was reverse transcribed under conditions recommended by the manufacturer (Agilent technologies).

### 2.4. Quantitative Polymerase Chain Reaction

The level of mRNA of UPR target genes, IRE1, ATF6, PERK, and XBP-1, has been assessed using real-time RT-PCR (Biorad, Marnes-la-Coquette, Charbonnières-les-Bains France). The PCR was performed using the primers shown in Table 1 and SYBR green PCR core reagents according to the manufacturer’s instructions (Biorad, Marnes-la-Coquette, Charbonnières-les-Bains, France). PCR conditions were as described previously [31].

The results were normalized to 18S rRNA expression levels [28]. Relative expression was evaluated with ΔΔCT method.

### 2.5. Cells Lysates

For immunoblot and ELISA assays, cells were washed twice with ice-cold phosphate buffer saline (PBS) and lysed at 4 °C for 20 min in PBS containing 1% NP40, 0.5% deoxycholic acid, and 0.1% SDS added with protease inhibitors (10 mM PMSF, 1 µM leupetin/pepstatin A, and 1 mg/mL of iodoacetamide). Protein concentrations were determined using the Micro BCA protein assay kit (Thermo Scientific, France).

### 2.6. Electrophoresis and Immunoblotting

Total cell extracts were prepared as described [30]. Protein samples were separated by SDS-PAGE and transferred to nitrocellulose membranes. The blots were probed using monoclonal anti-CFTR antibody (1 µg/mL), L12B4 (Millipore, France). Primary antibodies were visualized by horseradish peroxidase-conjugated sheep anti-mouse IgG and ECL detection Kit (Covalab, France). Immunoblotting of Na^+^/K^+^-ATPase α1-subunit was performed with the mouse monoclonal antibody (0.5 µg/mL; α6F, DSHB, University of Iowa).

### 2.7. Cell Viability Using MTT Test

Cells were cultured in 96-well plates upon confluency. Following the treatments, cells were incubated 4 h in phenol red free medium containing 10% of MTT ([4,5-dimethylthiazol-2-yl]- 2,5-diphenyl tetrazolium bromide; Sigma). The MTT solution was finally replaced by MTT lysis solution (10% Triton X-100 and 0.1 N HCl in anhydrous isopropanol), and the resulting optical density (DO) was determined after measurement of the difference between 570 nm and 690 nm absorbance.

### 2.8. Intracellular Copper Determination

To evaluate the intracellular copper concentration, cells were collected and stored at room temperature (RT), for at least 48 h, to allow them to completely dry and be used for ICP-AES analysis. Samples were then vacuum-dried and mineralized in 70% nitric acid before analysis. The Inductively Coupled Plasma-Atomic Emission Spectrometry (ICP-AES) with a Variant, Vista MPX instrument was used. The copper (Cu), iron (Fe), and zinc (Zn) contents were reported in relation to the cells number (nmol/10^6^ cells).

### 2.9. ROS Measurements

Cells (10^6^ cells/well) were seeded in a 24-well plate and cultured for 24 h. The cells were washed and changed to serum-free media and incubated with 50 μM 5-(and 6)-chloromethyl-2′,7′-dichlorodihydrofluorescein diacetate acetyl ester (H2DCFDA; Thermofisher, Saclay, Charbonnières-les-Bains, France) for 45 min. The conversion of H2DCFDA to fluorescent DCF was measured using a plate reader Infinte M200 (TECAN, France). DCF fluorescence was measured at λex/λem of 495/527 nm.

### 2.10. Catalase Enzyme Activity Assays

The catalase activity was determined using a spectrophotometer at 240 nm, by measuring the decomposition of hydrogen peroxide (H_2_O_2_). The Beers and Sizer method was used [32]. Specific enzyme activity (SA) was expressed as units per mg of protein.

### 2.11. Cu/Zn- and Mn-Superoxide Dismutase (SOD) Activities

The total SOD activities, including Cu/Zn- and Mn-SOD, was measured according to the method of S. Marklund and G. Marklund based on the inhibition of pyrogallol (1,2,3- trihydroxybenzene, C_6_H_6_O_3_) autoxidation [33]. Absorbance was measured at 420 nm for 5 min. One unit of SOD activity is defined as the amount of enzyme that inhibits the rate of pyrogallol autoxidation by 50%. To determine Mn-SOD activity, the potassium cyanide (KCN) was added to inhibit the Cu/Zn-SOD reaction.

### 2.12. Glutathione Peroxidase (GPx) Activity

The GPx activity was determined according to the method of Flohe and Gunzler [34]. The reaction is based on the reduction of organic hydroperoxides to alcohols by GPx, along with the oxidation of reduced glutathione (GSH) to oxidized glutathione (GSSG). GSSG is then reduced by glutathione reductase (GR), in the presence of NADPH. The GPx activity in the samples is determined through a follow-up of the decrease in NADPH absorption at 340 nm for 3 min.

### 2.13. Mitochondrial Isolation

Isolation of mitochondria from cell pellets was achieved using a differential centrifugation procedure [35]. The pellet of bronchial cells was resuspended in 250 µL of mitochondrial isolation buffer (210 mM mannitol, 70 mM sucrose, 5 mM Tris-HCl, 1 mM EDTA, pH 7.5) and homogenized with 15 strokes in a Dounce homogenizer (Fisher Scientific, France) on ice. To obtain mitochondria, the homogenate was subjected to differential centrifugation steps as described previously [35].

Mitochondrial purity and efficacy of isolation were assessed by the determination of lactate dehydrogenase (LDH) and glutamate dehydrogenase (GDH) activities.

### 2.14. GDH and LDH Activities

To assess mitochondrial fraction contamination by cytosolic components, we measured LDH and GDH activities in the mitochondrial and cytosolic fractions [36]. LDH and GDH are two enzymes that are exclusively found in the cytosol and mitochondria, respectively. Before LDH and GDH analyses, the mitochondrial pellets were resuspended in PBS and lysed by the addition of lauryl dimethylamine *N*-oxide to a final concentration of 0.3% (vol/vol). LDH activity in both fractions was determined kinetically by monitoring the loss of NADH at 340 nm for 10 min. GDH activity was determined as previously reported [37]. The assay quantified GDH activity based on the consumption of NADH in the transamination of α-ketoglutarate (oxoglutarate) monitored at 340 nm. Purity and isolation efficiency were expressed as the percentage of the total activity of LDH and GDH in the samples.

### 2.15. Aconitase and Fumarase Activity Assays

Before the determination of the aconitase activity, freshly isolated mitochondria were suspended in 0.5 mL of buffer containing 50 mM Tris-HCl (pH 7.4) and 0.6 mM MnCl_2_ and sonicated for 2 s. Aconitase activity was measured using a spectrophotometer by monitoring the formation of cis-aconitate from added iso-citrate (20 mM) at 240 nm and 25 °C. Fumarase activity was determined by measuring the increase in absorbance at 240 nm at 25 °C in the reaction mixture to which 30 mM potassium phosphate (pH 7.4), and 0.1 mM L-malate were added. Aconitase and fumarase activities were expressed as units per µg of protein.

### 2.16. Cytokine Secretion by Sandwich ELISA

Interleukins (IL) 1β, IL-6, IL-8, IL-10, IL-17 (A, E, F) and tumor necrosis factor-alpha (TNFα) released into the culture media and present in proteins extract were assayed using a quantitative sandwich enzyme-linked immunoassay kit (R&D Systems). According to the manufacturer, the sensitivity of this assay system is less than 10 pg/mL.

For ELISA assays used of this study, very low auto-fluorescence was detected in the absence of any treatment. The value of this auto-fluorescence was systematically subtracted from the experimental values.

### 2.17. Statistical Analysis

Differences between mean values were compared by Student’s unpaired two-tailed *t*-tests using SigmaStat (Jandel Scientific Software, SanRafael, CA, USA). Data are expressed as mean ± SEM, unless otherwise indicated. Significance was set at a two-tailed *p* value of 0.05.

## 3. Results

### 3.1. Bronchial Epithelial ROS Production Is Independent of CFTR Expression and Function

CF airway epithelial cells and neutrophils exhibit constitutive oxygen-derived reactive oxygen species (ROS) generation that leads to irreversible lung damage [38,39]. However, the role of CFTR in bronchial epithelial ROS generation is still not clear. Using the well-characterized transformed healthy (HBE) and CF (CFBE) bronchial epithelial cells, we determined the role of CFTR in ROS production.

**Figure 1:**
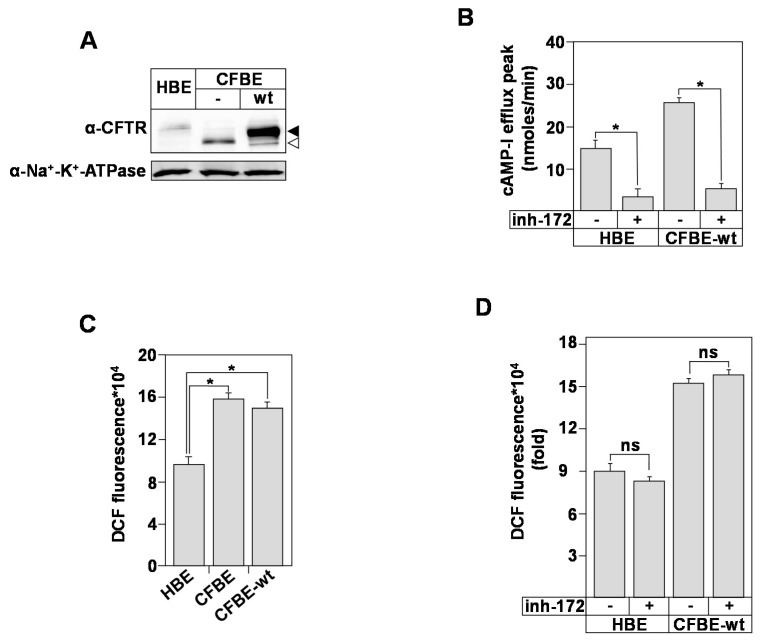
Characterization of CF Transmembrane Conductance Regulator (CFTR) expression and reactive oxygen species (ROS) production. (**A**) Expression of CFTR protein normalized to Na^+^/K^+^-ATPase in human bronchial epithelial cells; control (healthy bronchial epithelial) (HBE), cystic fibrosis (CFBE), and CFBE stably transfected with wild (wt) type CFTR (CFBE-wt). Black arrow corresponds to fully glycosylated mature CFTR form (band C). White arrow corresponds to core-glycosylated CFTR (band B). (**B**) Iodide efflux used as a functional assay to determine the activity of CFTR chloride channel in HBE, CFBE, and CFBE-wt cells, in the absence or presence of 10 µM of inh-172. (**C**) Measurement of ROS concentration in HBE, CFBE, and CFBE-wt cells using dichlorodihydrofluorescein (DCF) fluorescent probe. Cells were incubated with 50 μM dichlorodihydrofluorescein diacetate acetyl ester (H2DCFDA) for 45 min. The conversion of H2DCFDA to fluorescent DCF was measured at λex/λem of 495/527 nm. (**D**) Measurement of ROS concentration in HBE and CFBE-wt, in the absence or presence of inh-172 using the DCF fluorescent probe. Values with an asterisk are significantly different from their corresponding controls (*p* < 0.05). ns: nonspecific. Data are expressed as mean ± SEM (*n* = 6).

First, we characterized the CFTR expression and function in the bronchial epithelial cell model. Using Western blot analysis (Figure 1A) and image J quantification (Figure S1A), we detected CFTR protein in HBE, CFBE, and CFBE-wt protein extracts. The analysis showed the presence of the complex- (black arrow) and the core- (white arrow) glycosylated forms. Our results also confirmed the presence of the complex-glycosylated CFTR in CFBE-wt cells resulting from the stable expression of wt-CFTR (Figure 1A). Compared to HBE and CFBE-wt cells, the CFBE express only the core-glycosylated form (Figure 1A, white arrow). Functional assays confirmed the presence of CFTR at the plasma membrane of HBE and CFBE-wt cells (Figure 1B). CFTR Cl^–^ activity, measured using cAMP-activated I efflux, was inhibited in both cell lines in the presence of CFTR-inh-172 (inh-172), a potent and very specific inhibitor of CFTR Cl^–^ channel [29] (Figure 1B).

To further analyze the relationship between ROS production, CFTR expression, and CFTR Cl^–^ function, the intracellular ROS variations were monitored. Using a ROS-sensitive fluorescent probe (H2DCFDA), we observed that the intracellular levels of ROS were higher (~two fold) in CFBE cells compared to HBE (Figure 1C). Correction upon the expression of wt-CFTR (CFBE-wt) did not attenuate the level of ROS in CFBE cells (Figure 1C). In addition, the inhibition of the CFTR Cl^–^ activity, using the CFTRinh-172 (inh-172) did not affect ROS levels neither in HBE nor in CFBE-wt cells (Figure 1D), suggesting that CFTR channel’s activity is not involved in ROS production.

Our results showed that ROS levels in CFBE cells are not influenced by the expression or the activation of CFTR Cl^–^ channel. These observations also suggested that CFTR-independent mechanisms might participate in the CF-associated oxidative stress (OX-S).

To better evaluate the OX-S in CFBE compared to HBE and CFBE-wt cells, we assessed various causes that have been associated with cell OX-S, including ER stress, mitochondria metabolism, antioxidant enzymes activities, and deregulations in the status of essential trace elements such as copper (Cu), iron (Fe), and zinc (Zn), [24,25,26,27].

### 3.2. Antioxidants Enzymes Activities in the Bronchial Epithelial Cells

First, we measured the activities of the cytosolic superoxide dismutases (SOD) Cu/Zn form (Cu/Zn-SOD) and the mitochondrial SOD manganese form (Mn-SOD).

**Figure 2:**
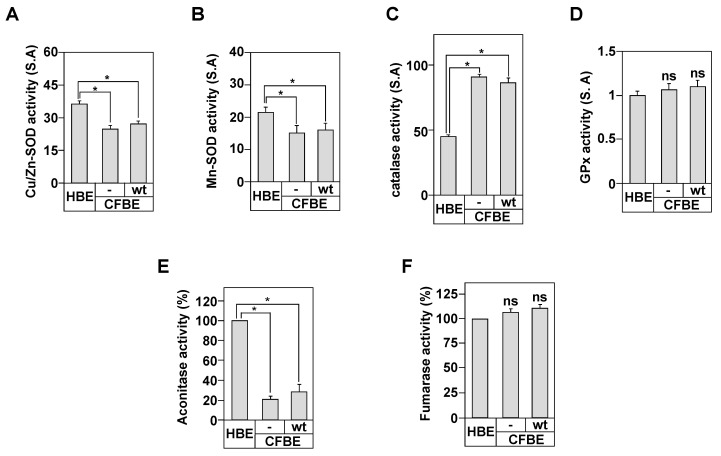
Characterization of the oxidant/antioxidant system in CF bronchial cells. Cu/Zn-SOD (**A**), Mn-SOD (**B**), catalase (**C**), glutathione superoxydase (GPx) (**D**), aconitase (**E**), and fumarase (**F**) activities were measured in HBE, CFBE, and CFBE-wt cells as described in Materials and Methods. One unit of the total SOD activity was defined as the amount of enzyme that inhibits the rate of pyrogallol autoxidation by 50%. The absorbance was measured at 420 nm for 5 min. Catalase activity was determined at 240 nm by measuring the decomposition of hydrogen peroxide (H_2_O_2_). GPx activity in the samples was determined by monitoring the decrease in NADPH absorption at 340 nm for 3 min. Aconitase activity was measured by monitoring the formation of cis-aconitate upon addition of iso-citrate (20 mM), at 240 nm and 25 °C. Fumarase activity was determined by measuring the increase in the absorbance at 240 nm at 25 °C. Values with an asterisk are significantly different from their corresponding controls (*p* < 0.05). Data are expressed as mean ± SE (*n* = 6).

Both enzymes are the first line of defense against the deleterious effects of ROS [33]. Our results demonstrate that both activities are decreased in CFBE compared to HBE cells (Figure 2A,B). Interestingly, the stable expression of wt-CFTR in CFBE cells did not correct this defect, suggesting that both activities are independent from CFTR expression. Because SOD activity scavenges superoxide anions (O_2_^−^) and converts them to hydrogen peroxide (H_2_O_2_) [32,40], a molecule that is transformed by the catalase and glutathione peroxidase (GPx) enzymes, we measured the activity of both enzymes in our cell model.

The catalase activity was almost two-fold higher in CFBE compared to HBE cells, confirming the over-production of CF-associated ROS (Figure 2C). However, the catalase activity did not decrease following the correction by wt-CFTR protein (Figure 2C). No change in GPx activity was depicted in CFBE cells compared to HBE and CFBE-wt (Figure 2D), suggesting a limited role of GPx in CF-associated OX-S.

### 3.3. In Vitro Markers of Mitochondrial Oxidative Stress

Mitochondria are the primary site of H_2_O_2_ generation by aerobic metabolism [40]. At the mitochondrial level, the Mn-SOD is responsible of the elimination of H_2_O_2_ generated by the electron transport chain, which protects the mitochondria from oxidative damage [40].

Because the activity of Mn-SOD was reduced in CFBE cells (Figure 2B), we determined the mitochondria function in the context of CF disease and in relation to CFTR protein.

First, we determined the purity of our mitochondrial fraction prepared from HBE, CFBE, and CFBE-wt cells by measuring LDH and GDH activities, indicators of the cytosol contamination and mitochondrial enrichment, respectively [35]. We could recover up to ~85.7 ± 2.5% of the total GDH activity detected in HBE, CFBE, and CFBE-wt homogenates (Figure S1B). The remaining activity was present in the cytosolic fraction. For LDH, only ~5.7 ± 0.8% of the total activity was detected in the mitochondrial fractions (Figure S1C). These results indicate that the mitochondrial fractions are relatively free of cytosolic contamination. The mitochondrial fraction was then used to measure the oxidative stress through the assessment of the activity of two enzymes, the aconitase that is inactivated by oxidant molecules and fumarase, an enzyme that is unaffected by such stimuli [35]. Our results show that mitochondria from CFBE cells exhibited 80% lower aconitase activity compared to HBE cells (Figure 2E). The loss of aconitase activity in CFBE cells was independent of CFTR expression since similar loss in CFBE-wt cells was observed (Figure 2E). Mitochondrial fumarase activity in CFBE, CFBE-wt, and HBE cells was unchanged (Figure 2F). These results support the conclusion that CF bronchial epithelial cells have an intrinsic OX-S that appears to be independent from the CFTR expression and function.

### 3.4. In Vitro Indicators of Endoplasmic Reticulum (ER) Stress

ER stress and UPR (Unfolded Protein Response) activation are central to the stimulation of cellular inflammation [41,42]. Yet, the relationship between CFTR expression in bronchial cells and ROS production is unknown.

**Figure 3:**
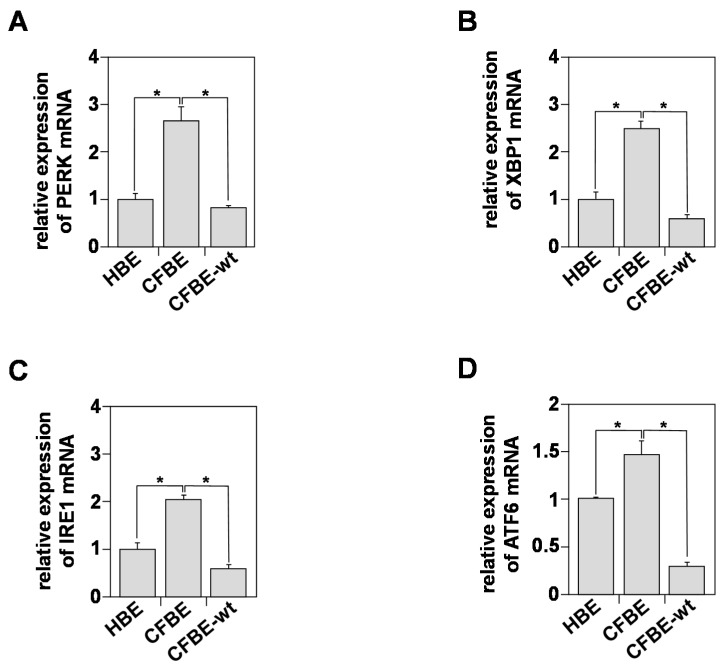
Unfolded Protein Response (UPR) characterization in healthy and CF bronchial cells. Relative mRNAs expression of PERK (**A**), XBP1 (**B**), IRE1 (**C**), and ATF6 (**D**) in HBE, CFBE, and CFBE-wt cells using real-time RT-qPCR. 18S rRNA expression was used as an internal control. Values overwritten with stars are significantly different from the control (*p* < 0.05). Data are expressed as mean ± SE (*n* = 6).

UPR is mediated by the activation of three ER transmembrane stress sensors: IRE1 (inositol-requiring transmembrane kinase/endonuclease-1); PERK (PKR-like ER kinase); and ATF6 (activating transcription factor) [41,42]. We compared the levels of expression of IRE1, PERK, ATF6, and XBP-1 (X-box binding protein 1). The latter is a transcription factor that regulates the expression of genes important for the cellular inflammation and stress responses [42]. Figure 3A,D show that the levels of the three proteins were significantly elevated in CFBE cells compared to HBE.

The levels of PERK (Figure 3A), XBP-1 (Figure 3B), IRE1 (Figure 3C), and ATF6 (Figure 3D) were ~2.5, ~2.3, ~2, and ~0.5 fold higher in CFBE compared to HBE cells, respectively. These results demonstrate the presence of an intrinsic UPR activation process in CFBE cells that is independent of exogenous stimuli, such as infection or inflammation. Interestingly, restoring plasma membrane CFTR expression and Cl^–^ secretion reversed the activation of UPR, suggesting a relationship between CFTR and UPR in bronchial epithelial cells.

### 3.5. Inflammatory Profile of Healthy and CF Bronchial Epithelial Cells

**Figure 4:**
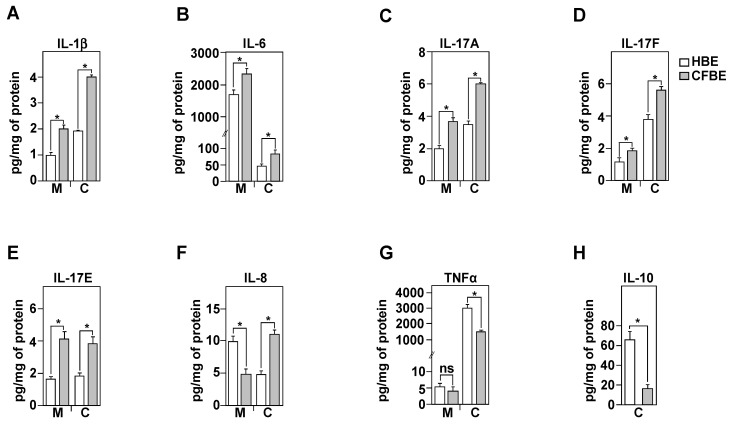
Evaluation of inflammatory profile in healthy and CF bronchial cells. Measurements of IL-1β (**A**), IL-6 (**B**), IL-17A (**C**), IL-17F (**D**), IL-17 (**E**), IL-8 (**F**), TNFα (**G**), and IL-10 (**H**), released in the culture media (M), or present in protein extracts (C). The cytokines were assayed using a quantitative sandwich enzyme-linked immunoassay (ELISA) in HBE (white charts) and CFBE (black charts). Concentrations are in pg/mg of extracted proteins. Values overwritten with asterisk are different from the control (*p* < 0.05). Data are expressed as mean ± SE (*n* = 6).

Because exacerbated ROS production in CFBE cells (Figure 1C) may activate various signaling pathways that increase the production and secretion of pro-inflammatory cytokines, in the absence of any bacterial or viral infections, we analyzed the steady state inflammatory profiles of healthy (HBE) and CF (CFBE) bronchial epithelial cells. Using ELISA assays, we evaluated both the production (C; for cell) and secretion (M; for medium) of the pro- (IL-1β, IL-8, IL-6, IL-17A, IL-17F, IL-17E, TNFα) and the anti-inflammatory (IL-10) cytokines. All these cytokines were reported to be deregulated in CF, particularly in the bronchoalveolar lavages (BAL), fluids collected from CF patients [17]. Compared to HBE, the CFBE cells showed a significant increase in the production and the secretion of IL-1β, IL-6, IL-17A, IL-17F, and IL-17E (Figure 4A–E). However, compared to HBE cells, CFBE exhibited a significant decrease in IL-8 secretion despite its capacity to produce it at high levels (Figure 4F). These results suggest that the CFBE cells may have a defective secretion/exocytosis system linked to the delF508 mutation.

Compared to HBE cells, our results also demonstrate a significant decrease in the production of the TNFα in CFBE cells, while its levels of secretion remained unchanged (Figure 4G). Finally, no IL-10 secretion was detected in the culture medium. This was not due to a limitation in the detection as we could measure this cytokine within the cells at value around 10 pg/mL in CFBE and ~63 pg/mg of protein in HBE cells (Figure 4H).

### 3.6. IL-8 Secretion Is Associated with CFTR Function

**Figure 5:**
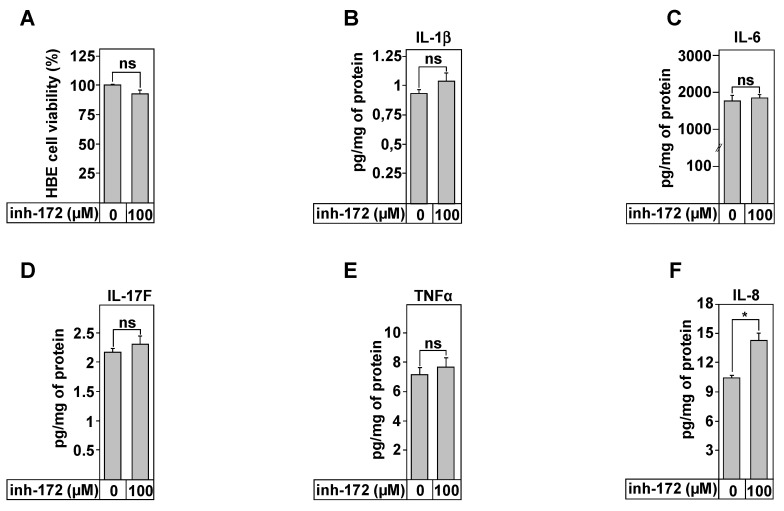
Effect of CFTR inhibition on cytokines secretion in HBE cells. (**A**) Evaluation of HBE viability using MTT assays following 24 h incubation with 100 µM of inh-172, a CFTR chloride channel inhibitor. ELISA tests were used to measure IL-1β (**B**), IL-6 (**C**), IL-17F (**D**), TNFα (**E**), and IL-8 (**F**) secretion in the culture media of HBE cells treated during 24 h with 100 µM of inh-172. Values overwritten with stars are significantly different from the control (*p* < 0.05). Data are expressed as mean ± SE (*n* = 6).

The data regarding the role of CFTR function in the control of the inflammatory profile of CF lung epithelial cells is very conflicting and depends on the protocol, on the cell types used, and on culture conditions [17]. In our study, we used HBE cells known for their ability to develop a highly polarized cell layer. To determine the effect of CFTR inhibition on the secretion of pro-inflammatory cytokines, HBE cells were treated with CFTRinh-172 (Figure 5). We first tested the effect of the drugs on the cell viability, since CFTRinh-172 was reported to disturb the mitochondrial function in HELA cells [43]. Dose- and time-dependent experiments are reported (Figure S2). The summary of these results is reported on Figure 5A. Compared to untreated HBE cells, even 100 µM of CFTRinhi-172 was without effect on cell viability, excluding any apoptotic effect of this molecule (Figure 5A). The inflammatory profile was therefore evaluated, in the absence and in the presence of CFTRinh-172. The results show that a treatment for 24 h did not affect the levels of secretion of IL-1β, IL-6, IL-17F, and TNFα (Figure 5B–E). However, the secretion of the pro-inflammatory cytokine, IL-8, was significantly increased from 9.7 ± 0.2 (control) to 13.3 ± 0.4 pg/mg of protein (Figure 5F).

### 3.7. Homeostasis of Bioactive Trace Metals Is Dysregulated in CF Bronchial Epithelial Cells

**Figure 6:**
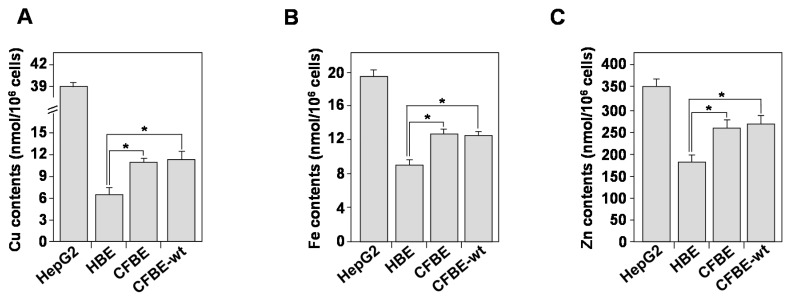
Inductively Coupled Plasma-Atomic Emission Spectrometry (ICP-AES) determination of biometals concentrations in healthy and CF bronchial cells. (**A**) Copper (Cu), (**B**) Iron (Fe), and (**C**) Zinc (Zn) content was assessed in HBE, CFBE, and CFBE-wt cells. For the control, the hepatocyte cell line (HepG2) was used as a positive control. Values overwritten with stars are significantly different from the control (*p* < 0.05). Data are expressed as mean ± SE (*n* = 6).

In addition to UPR and mitochondria dysfunction, intracellular biometals, such as zinc (Zn), copper (Cu), and iron (Fe), may contribute to cellular ROS production [44]. Increased metal levels may undergo a Fenton reaction and form highly toxic hydroxyl radicals (•OH) that induce an OX-S [44]. To compare the intracellular concentration of Cu, Zn, and Fe in HBE, CFBE, and CFBE-wt cells, we used the ICP-AES analysis. Hepatocyte cell line HepG2 was used as a positive control to validate the measurement, as these cells are known to participate in metal detoxification [45]. Compared to HBE cells, the intracellular concentrations of Cu (Figure 6A), Fe (Figure 6B), and Zn (Figure 6C) were significantly increased. The introduction of wt-CFTR did not correct the biometals deregulation in CFBE cells (Figure 6A–C), suggesting that other mechanisms or other proteins might be involved in the CF trace elements dyshomeostasis.

The increase in the intracellular concentration of Cu, Fe, and Zn in CFBE cells may be the consequence of the retention of CFTR-dF508 in the ER compartment. To verify this hypothesis, we measured the intracellular concentration of these elements in BHK (baby hamster kidney) and BHK cells overexpressing CFTR-dF508 (BHK-dF). Figure S3 shows that CFTR-dF508 did not affect the intracellular levels of Cu, Fe, and Zn. Additionally, the overexpression of wild type CFTR in CF-IB3 cells to generate SF9-cell line did not affect the levels of these elements (Figure S3).

### 3.8. Effect of Copper Treatment on Healthy and CF Inflammation Responses

The above results demonstrated that numerous processes that are linked to copper homeostasis were deregulated in CF cells; these include decreased Cu/Zn- and Mn-SOD activities, deregulated mitochondrial activity, and increased intracellular Cu contents. Additionally, previous data showed decreased cytoplasmic and mitochondrial concentration of glutathione, a potent Cu chelator in CF lung epithelial cells [21]. Based on these findings, we hypothesized that Cu is a biometal that may play a critical role in CF, particularly in relation to OX-S and inflammation.

**Figure 7:**
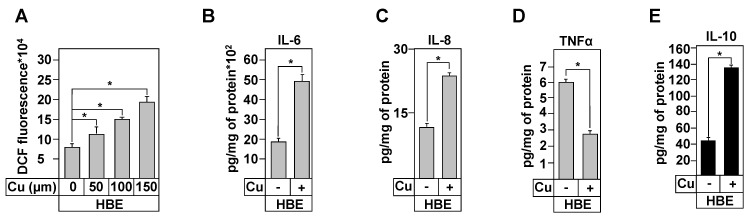
Effect of copper treatment on the inflammation profile of healthy and CF bronchial cells. (**A**) Measurement of ROS levels in HBE cells following treatment with the indicated copper (Cu^2+^) concentrations. Cells were incubated with 50 μM H2DCFDA for 45 min. The conversion of H2DCFDA to fluorescent DCF was measured at λex/λem of 495/527 nm. Measurement of IL-6 (**B**), IL-8 (**C**), TNFα (**D**), and IL-10 (**E**) concentrations in the culture media (grey chart) and in protein extracts (black chart) of HBE cells after treatment with 100 µM of Cu, during 24 h. Cytokine levels were assayed using ELISA tests. Concentrations are in pg/mg of protein extracts. Values overwritten with stars are significantly different from the control (*p* < 0.05). Data are expressed as mean ± SE (*n* = 6).

To verify this hypothesis, we incubated HBE cells for 24 h in the presence of copper. First, we showed that copper treatment increased intracellular copper concentration (data not shown, [28]). We then measured ROS production following copper treatment. Figure 7A shows that incubation with Cu increased ROS production in a dose-dependent manner. This finding suggests that elevated extracellular copper levels, as reported in BAL of CF patients [46], may induce OX-S, one of the triggers of local inflammation.

We then determined the inflammatory responses of HBE cells, in the absence or presence of copper (100 µM). Figure 7B,C show that copper treatment increased IL-6 and IL-8 secretion and IL-10 production (Figure 7E). Secretion of TNFα was significantly deceased (Figure 7D) and no change was observed in the levels of IL-1β and IL-17 inflammatory cytokines (Figure S4). These data strongly suggest that copper is involved in OX-S-mediated inflammation in CF cells

Previous data from our group demonstrated that lower concentrations of intracellular copper in trophoblast cells also increased ROS production [31]. To test this hypothesis in lung cells, we treated HBE cells with the specific extracellular copper chelator, bathocuproine sulphonate (BCS), and determined their viability; the levels of intracellular copper concentration; and ROS production and the levels of secreted cytokines.

No changes in these parameters were observed, Figures S5 and S6, suggesting that the chelation of the extracellular copper using BCS does not influence the intracellular copper concentrations, most of which is in a bounded form.

## 4. Discussion

In the present study, we demonstrate that bronchial epithelial cells homozygous for the delF508 mutation exhibit a constitutive secretion of pro-inflammatory cytokines and an intrinsic oxidative stress response. These two processes were evaluated in the absence of bacterial infection, a process thought to be the earliest step in CF development [10,11,12]. While the study was based on in vitro findings that mainly used the HBE cell model, it allowed advancing our understanding on the CF disease. In fact, this cell type represents the first line of defense against the external infections [47] and has the advantage of developing a highly polarized cell layer compared to other previously used cell lines [28]. ROS production in CF cells was independent of CFTR mutation, expression, or function, confirming previously reported data that used other cellular models [48,49]. Our results also demonstrate the decrease in the antioxidant response that was not corrected by the expression of CFTR-wt protein.

For the first time, we demonstrate that the activity of Mn-SOD, an important enzyme of mitochondrial function, was decreased in CF bronchial cells in a CFTR-independent manner. This result is in line with the decrease in the aconitase activity, an enzyme associated with mitochondrial function, suggesting that the local production of H_2_O_2_ may not be eliminated by Mn-SOD, which may contribute in the long-term accumulation of the intracellular ROS in CF cells.

Another parameter involved in the production of ROS is the UPR system [50]. We demonstrated that the UPR activity is detected in CF bronchial epithelial cells in a CFTR-dependent manner. This finding is in line with previously published studies, showing that correction of CFTR-delF508 trafficking decreased UPR activation [51].

We also provided evidences that the OX-S is responsible for the inflammation responses that characterize CFBE cells. However, some discrepancies were observed for IL-8, as these cells only produced IL-8 but could not secrete this cytokine. This lack of secretion has also been observed in other studies using primary culture models [52]. Importantly, IL-8 was increased upon the inhibition of CFTR, suggesting a potential side effect of the inh-172 molecule as previously observed in Hela and IB3 cells [43]. The most intriguing result was the loss of the production and secretion of the anti-inflammatory cytokine IL-10 by CFBE cells, suggesting that other processes may be involved in the bronchial intrinsic CF inflammatory responses.

In addition to UPR and mitochondria dysfunction, intracellular biometals such as zinc (Zn), copper (Cu), and iron (Fe) may undergo a Fenton reaction that contributes to cellular ROS production [44]. For the first time, we demonstrate that CF cells exhibit high intracellular concentrations of copper, iron, and zinc that were independent from CFTR.

The existence of such deregulations was also documented in the BAL of CF patients [53]. However, the underlying mechanisms are still to be demonstrated. Because the CF OX-S has been associated with alterations in oxidant/antioxidant actors [25,26], which are mainly related to copper for their activities, [27,46], we only focused on copper effect on the inflammation responses. Upon copper treatment, only pro-inflammatory cytokines secretion was disturbed, suggesting that copper deregulations in CF patients might trigger the inflammatory responses through the activation of OX-S. Nevertheless, a link between the cupric status in CF and the activation of the master regulator of the pro-inflammatory cytokines [13], the NFκB, is still missing.

The link between copper and inflammation and the correlation between oxidative stress and ROS production provide strong evidence that support the idea that ROS production by the CFBE cells may contribute to the activation of inflammatory process.

This work uncovers the intrinsic inflammatory and oxidative stress responses that are activated at the onset of the CF disease, in the absence of any infection and/or pro-inflammatory stimuli. The identification of cellular copper as a key factor in the generation of CF-associated OX-S opens new areas of investigation to better understand CF-associated early inflammation.

## Figures and Tables

**Table 1:** Primers used for real-time RT-PCR. FW: Forward, BW: Backward (reverse primer).

Gene	Primer Sequence 5′→3′	Size
PERK	FW: TCTGTTCAGCTCTGGGTTG TBW: CCGAAGTTCAAAGTGGCCAA	158 bp
XBP-1	FW: TGTCACCCCTCCAGAACATC BW: AAGGGAGGCTGGTAAGGAAC	196 bp
IRE1	FW: AGCAAGAGGACAGGCTCAAT BW: CATCTGAACTTCGGCATGGG	205 pb
ATF6	FW: GTGTCAGAGAACCAGAGGCT BW: GGTGCCTCCTTTGATTTGCA	166 bp

## Data Availability

Data available in a publicly accessible repository.

## Supplementary Materials

The following are available online at https://www.mdpi.com/2227-9059/9/4/329/s1.
