# Supplementary material for: Copper-Associated Oxidative Stress Contributes to Cellular Inflammatory Responses in Cystic Fibrosis
Source: Biomedicines. 2021 Mar 24;9(4):329. doi: 10.3390/biomedicines9040329 (PMC8064106; doi:10.3390/biomedicines9040329)
Supplement: Supplementary file 1 [file biomedicines-09-00329-s001.pdf]

Type of the Paper: Article

Copper associated Oxidative Stress contributes to inflammatory responses in Cystic Fibrosis

Amal Kouadri[1,2,3§], Johanna Cormenier[1,2,3§], Kevin Gemy[1,2,3], Laurence Macari[1,2,3], Peggy Charbonnier[2,3,4], Pierre Richaud[5], Isabelle Michaud-Soret[2,3,4], Nadia Alfaidy[1,2,3], and Mohamed Benharouga[1,2,3].

[1]Institut National de la Santé et de la Recherche Médicale (INSERM), U1292, Laboratoire de BioSanté, Grenoble, France.

[2]Commissariat à l'Energie Atomique (CEA), DSV-IRIG, Grenoble, France.

[3]Université Grenoble Alpes (UGA), Grenoble, France.

[4]Centre National de la Recherche Scientifique (CNRS), LCBM-UMR 5249, Grenoble, France.

[5]Univ. Aix-Marseille, CNRS, CEA, Institut de Biosciences et Biotechnologies d'Aix-Marseille (BIAM), UMR 7265, CEA Cadarache, Saint-Paul-lez Durance F-13108, France.

§ These authors contributed equally to this study.

Address correspondence to:

Dr. Mohamed Benharouga

Biosanté-U1292, MAB2

DRF-IRIG, DS, CEA-Grenoble

17 rue des Martyrs, F-38054, Grenoble cedex 09, France

Téléphone: (+33) 4-38-78-44-51

E-mail: mohamed.benharouga@cea.fr

Supplementary Figures and Legends

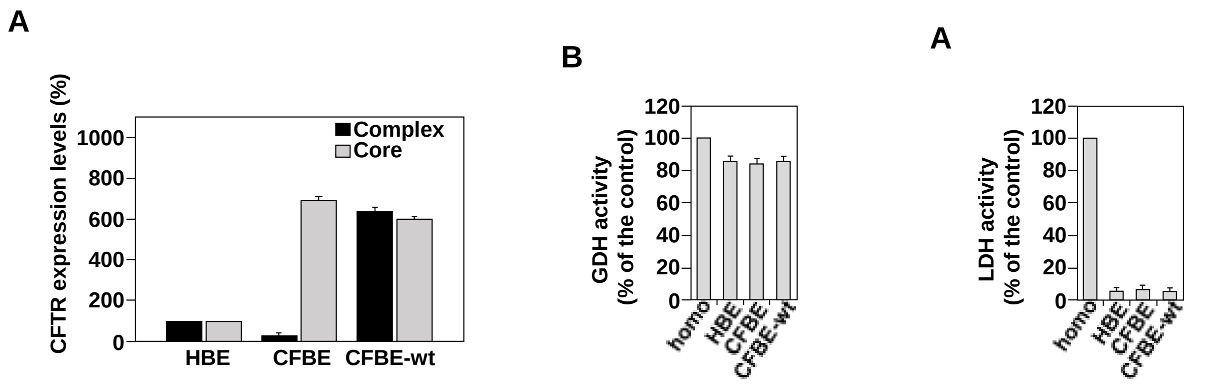

Figure S-1: Quantification of CFTR expression and determination of LDH and GDH activities in HBE, CFBE and CFBE-wt cells. (**A**) Quantification of the expression of CFTR complex- and core-glycosylate form using Image J. (**B**) GDH activity was determined by monitored at 340 nm the decrease of NADH concentration during transamination of the α-ketoglutarate (oxoglutarate). (**C**) LDH activity was determined by monitoring the loss of NADH at 340 nm for 10 min. Data are expressed in % of the control as mean ± SE (n=6). "Homo" correspond to homogenate that was used as control.

Figure S-2

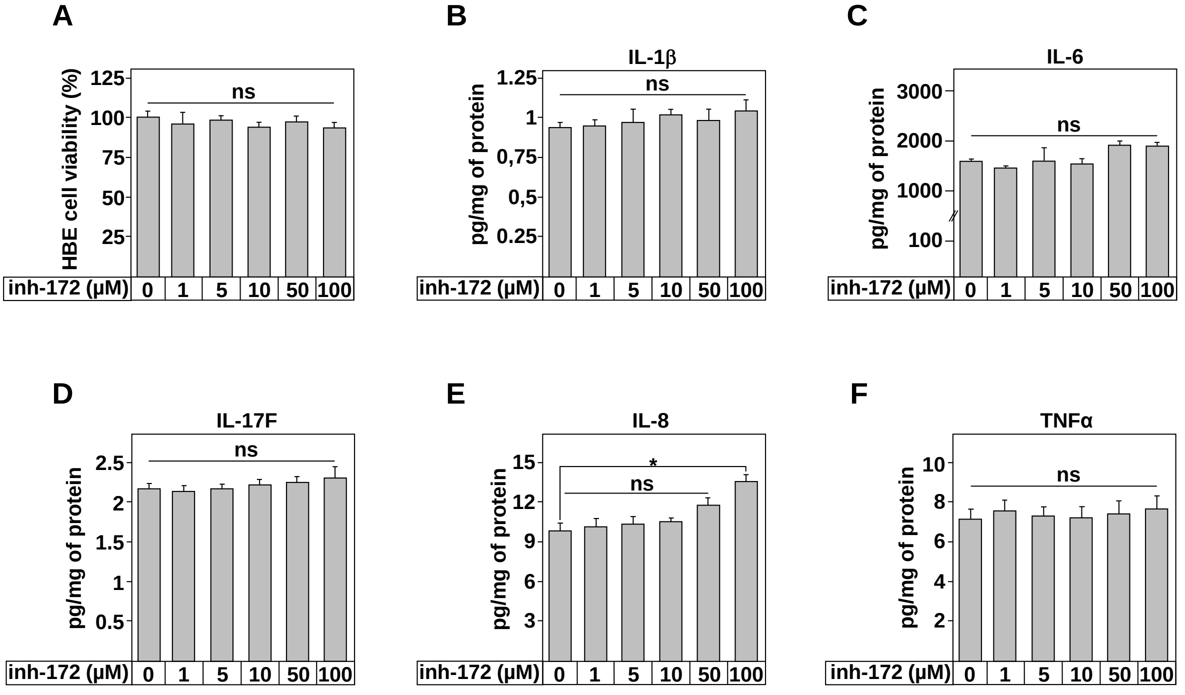

Figure S-2: Effects of concentration-dependent treatment of HBE cells with inh-172.

(**A**) Evaluation of HBE viability using MTT assays following 24h incubation with the indicated concentration of inh-172, a CFTR chloride channel inhibitor. Measurement of IL1β (**B**), IL6 (**C**), IL17F (**D**), IL8 (**E**), and TNFα (**F**) concentrations in the culture media (grey chart) of HBE cells after treatment with the indicated concentrations of inh-172 during 24h. Cytokine levels were assayed using ELISA tests. Concentration are in pg/mg of protein extracts. Values overwritten with stars are significantly different from the control ($P < 0.05$). Data are expressed as mean ± SE (n=6). ns correspond to non-significant.

Figure S-3

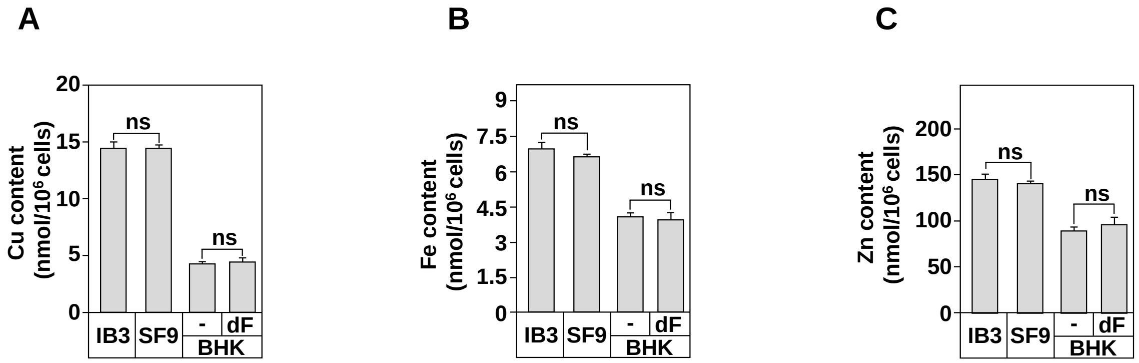

Figure S-3: ICPM-MS determination of biometals concentrations in IB3, SF9, BHK and BHK-dF. (**A**) Copper (Cu), (**B**) Iron (Fe), and (**C**) Zinc (Zn) content was assessed in IB3, SF9, BHK and BHK cells stably expressing CFTR-delF508 (BHK-dF). The content was determined using the inductively coupled plasma mass-spectrometry (ICP-MS) technique. Values overwritten with stars are significantly different from the control (P < 0.05). Data are expressed as mean ± SE (n=6). ns correspond to non-significant.

Figure S-4

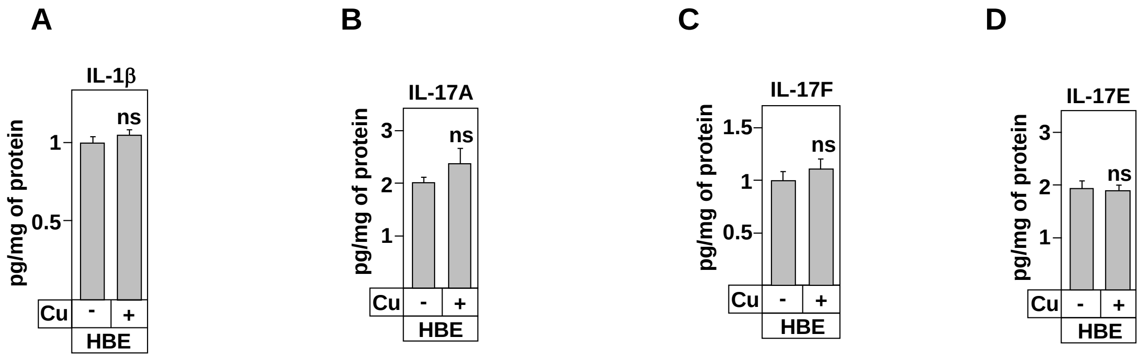

Figure S-4: Effect of copper treatment on the secretion of IL1β and IL17 (A, F, E) from HBE cells. Measurement of IL1β (**A**), IL17A (**B**), IL17F (**C**), and IL17E (**D**) concentrations in the culture media (grey chart) of HBE cells after treatment with 100 μM of Cu, during 24h. Cytokine levels were assayed using ELISA tests. Concentration are in pg/mg of protein extracts. Values overwritten with stars are significantly different from the control ($P < 0.05$). Data are expressed as mean ± SE (n=6). ns correspond to non-significant.

Figure S-5

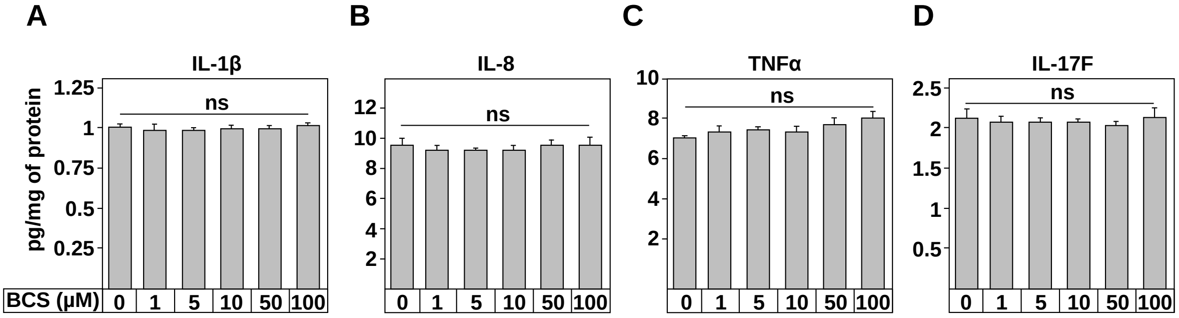

Figure S-5: Effect of BCS treatment on IL1β, IL8, TNFα, and IL-17F secretion from HBE cells. Measurements of IL1β (**A**), IL8 (**B**), TNFα (**C**) and IL17F (**D**) released into the culture media (grey chart) of HBE cells after treatment with the indicated concentration of BCS, a copper chelator, during 24h. Cytokines were assayed using ELISA tests. Values overwritten with stars are significantly different from the control ($P < 0.05$). Data are expressed as mean ± SE (n=6). ns correspond to non-significant.

Figure S-6

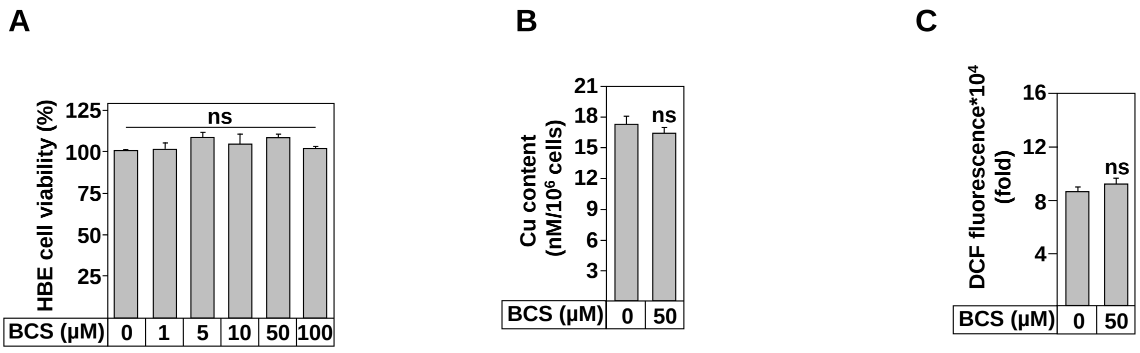

Figure S-6: Evaluation of BCS effect on HBE cells viability, copper content and ROS production. Determination of cell viability (**A**), copper content (**B**) and DCF fluorescence (**C**) in HBE cells after their treatment with the indicated BCS concentration.
